# Knowledge, Attitudes, and Concerns Regarding COVID-19 Vaccination Among Unvaccinated Elderly People in the Aseer Region, Saudi Arabia

**DOI:** 10.7759/cureus.42251

**Published:** 2023-07-21

**Authors:** Mohammed Y Asiri, Abdullah Alsabaani, Tariq A Falqi, Yahia M AlKhaldi, Abdullah Saeed, Nawal A Asiri, Mona S Alqahtani

**Affiliations:** 1 Research and Studies, Health Affairs Aseer Region, Abha, SAU; 2 Family and Community Medicine, King Khalid University, Abha, SAU; 3 Public Health, Health Affairs Aseer Region, Abha, SAU; 4 Family Medicine, Ministry of Health, Abha, SAU; 5 Action Research, Ministry of Health, Abha, SAU; 6 Nursing, Health Affairs Aseer Region, Abha, SAU

**Keywords:** unvaccinated, kingdom of saudi arabia (ksa), aseer region, elderly people, covid 19

## Abstract

Background: Vaccination is the best weapon to prevent any disease, especially pandemics like COVID-19, and building herd immunity is the best way to control the disease's spread. On one side, vaccine availability is important, and on the other, its successful distribution is faced with difficulty in a wide geographical area. Availability and distribution go hand in hand, which is the public health challenge. Vaccines are taking over, clearing up concerns about vaccination and making the public ready. The high-risk public should receive vaccines without resistance, which is of utmost importance. Unvaccinated older adults are at higher risk for COVID-19 infection, morbidity, and mortality. The reasons why older people who have not been vaccinated against COVID-19 do not want to receive vaccines when they are available include a lack of knowledge about COVID-19 vaccination and listening to incorrect sources of information. With this background, this study aimed to assess the knowledge, attitudes, and concerns of unvaccinated older adults in the Aseer Region, Saudi Arabia.

Methods: A community-based cross-sectional study was planned to find out the knowledge and attitudes of the elderly in the Assir Region, Saudi Arabia. A total of 434 unvaccinated elderly persons were recruited randomly from the list of elderly (>60 years) who were unvaccinated. The data was gathered using a structured interview schedule in Arabic.

Results: Out of 434 participants, more than half (54.8%) were male. Most of the participants were Saudi citizens (91.5%). Of the participants, nearly one-fifth (19.8%) of them had a previous history of COVID-19 infection. The participants' main sources of information about vaccination against COVID-19 were the mass media (41.9%), followed by word of mouth from friends, families, or neighbors (41.7%), and social media (16.4%). More than three-fourths of them (85.7%) had poor knowledge regarding the COVID-19 vaccination. The participants’ poor knowledge grades were mainly among those aged >80 years, illiterate, unemployed participants, and current smokers. Those participants relying on social media had the highest number of concerns (6.663.21) regarding the safety and efficacy of vaccines.

Conclusions: Most participants have poor knowledge, and their knowledge of COVID-19 vaccination is limited. Participants whose main source of knowledge is the mass media need to intensify their education activities in the mass media. Social media, whose primary source of information is social media, has the greatest number of issues that require immediate attention. Social media content must be scanned, and misinformation needs to be addressed.

## Introduction

China reported an exponential increase in new cases with symptoms ranging from cough, fever, and breathlessness to life-threatening conditions. On February 11, 2020, WHO identified the coronavirus as the cause of these symptoms. Later, it was named COVID-19 and declared a pandemic in March 2020 [1]. This COVID-19 pandemic has caused a heavy burden and a challenge to the public health system around the globe. COVID-19 has reached almost 220 countries across the globe; the WHO reported that more than 690.2 million individuals were infected, which killed almost 6.89 million people in the world. In the Kingdom of Saudi Arabia, nearly 700,000 people were infected, and almost 9,464 people lost their lives to COVID-19 [2]. Though the first case was reported two years ago, there is no definite antiviral treatment for COVID-19 [3,4].

To limit the community's spread of any communicable disease, the only way is through the development of herd immunity in the community. Vaccination is the critical approach to developing herd immunity, with adequate vaccine coverage and vaccine acceptance by the public. Vaccine acceptance depends on the public's beliefs and concerns about vaccines [5,6]. As per the Strategic Advisory Group of Experts on Immunization (SAGE), vaccine hesitancy is defined as a "delay in acceptance or refusal of vaccines despite the availability of vaccine services" [7-9].

The WHO persisted that the unwillingness or refusal to use vaccines would reverse progress in tackling vaccine-preventable diseases [10,11]. The public health administration in every country has planned to distribute vaccines to the public to end the COVID-19 pandemic. Governments, through mass media (e.g., radio, television), and non-governmental agencies like WHO are continuously working to build vaccine literacy among the public, which will make the public clear their concerns and accept vaccines when it is their turn. Rumors and misconceptions on social media about the safety and efficacy of vaccines create public fear and an unwillingness to undergo vaccination. These rumors will make it much more complicated to convince the public to accept vaccines. The Ministry of Health has spent much effort on these vaccine rumors and misconceptions circulating on social media to increase vaccine acceptance [12,13].

## Materials and methods

A community-based, cross-sectional study was conducted in the Aseer Region, Saudi Arabia between January and May 2022. Ethical approval was obtained from the Research Ethical Committee of the General Directorate of Health Affairs, Aseer Region, vide letter number RECNO: 1512020 dated January 24, 2022. The study participants were chosen at random from a list of unvaccinated COVID-19 elderly population; the list of unvaccinated and their mobile numbers was obtained from the Directorate of the Ministry of Health (MOH) in the Aseer Region. The minimum sample size was calculated based on previous studies reporting that 56% of the elderly refused COVID-19 vaccines. After substituting four pq/d2, the determined sample size was 378. The basic outline of the study tool was adapted from previous studies with modifications [14]. The study tool was prepared in the English language and was then translated into the local Arabic language and back-translated by two independent language experts to check the validity of the translation. The data were analyzed using SPSS Statistics version 21 (IBM Corp. Released 2012. IBM SPSS Statistics for Windows, Version 21.0. Armonk, NY: IBM Corp.). Consent was electronically obtained from participants who responded to the electronic online forms and verbally from illiterate participants, in accordance with the Declaration of Helsinki, after clearly explaining the objectives of this study to them.

## Results

A total of 434 elderly people responded. The study's elderlies were aged between 60 and 95 years, with a mean age of 72.2 years (± 0.4 years). Nearly half of the elderly, 216 (49.7%), were aged between 60 and 70 years. Of the 434 respondents, 196 (45.1%) were females and 238 (54.9%) were males. The difference between age groups and gender was not statistically significant (p >0.05). Most of the respondents, 397 (91.5%), were Saudi nationals, and 37 (8.5%) were non-Saudi Nationals. Out of the 434 respondents, more than half (243 or 56%) were illiterate; few were able to read and write (84 or 19.3%); nearly one quarter (101 or 23.3%) of the participants studied up to high school; and the remaining six (1.3%) completed their graduation. The difference between educational status and gender is statistically significant (p = 0.001). Most of the responders had one or more chronic diseases (372 (85.7%)), and the remaining 62 (14.3%) did not have any chronic diseases. The socio-demographic profile of the participants is shown in Table 1. In terms of perceptions of COVID-19 vaccines, nearly 45.2% (192) perceived that COVID-19 vaccines are not fake, and 39 (09%) perceived that COVID-19 vaccines are of no use at all. Almost 168 (39%) of the participants expressed that vaccines were developed very quickly and approved on an emergency basis. Nearly one-third (26.1%) of the participants perceived that COVID-19 vaccines were promoted commercially by big companies.

**Table 1:** Distribution of the study sample based on demographic variables BMI: body mass index

		Gender						
		Female		Male		Total		
		No	%	No	%	No	%	p-value
Nationality	Non-Saudi	0	0.0	37	15.5	37	8.5	
	Saudi	196	100.0	201	84.5	397	91.5	
Age group	60-70 years	87	44.4	129	54.2	216	49.8	p=0.15 (not significant)
	71-80 years	68	34.7	69	29.0	137	31.6	
	81-90 years	35	17.9	37	15.5	72	16.6	
	>90 years	6	3.1	3	1.3	9	2.1	
Marital status	Single	2	1.0	6	2.5	8	1.8	p=0.0001 (significant)
	Married	58	29.6	213	89.5	271	62.4	
	Divorced	35	17.9	5	2.1	40	9.2	
	Widow/widowed	101	51.5	14	5.9	115	26.5	
Occupation	Employed/retired	16	8.2	158	66.4	174	40.0	p=0.000 (significant)
	Unemployed	180	91.8	80	33.6	260	59.9	
Education	Illiterate	158	80.6	85	35.7	243	56.0	p=0.001 (significant)
	Literate	38	19.4	153	64.3	191	44.0	
Smoking	Yes	2	1.0	77	32.4	79	18.2	p=0.0001 (significant)
	No	194	99.0	161	67.6	355	81.8	
BMI	Underweight	3	1.5	5	2.1	8	1.8	p=0.63 (not significant)
	Normal weight	61	31.1	87	36.6	148	34.1	
	Overweight	81	41.3	90	37.8	171	39.4	
	Obese	51	26.0	56	23.5	107	24.7	

In terms of the COVID-19 infection status among the participants, around one-fifth of them, 86 (19.8%), were infected with COVID-19; perhaps the elderly thought that the natural immunity acquired through infection meant that they did not need vaccines. In addition, some of the elderly thought they were not infected even during the two peaks of COVID-19; thus, they had good immunity and did not need to take vaccines. Literates were infected slightly more than illiterates, 43 (22.5%), and almost equally males and females were infected, 47 (19.7%) and 39 (19.9%), respectively. The difference between education and infection status was not statistically significant (p>0.2). Nearly 22.8% (99) of them expressed that they were willing to receive COVID-19 vaccines, most of the elderly, 250 (57.6%), were not ready to take vaccines, and the remaining 85 (19.6%) were in a dilemma about whether to take them or not. Most of the participants, 170 (39.3%), prefer to acquire immunity against COVID-19 naturally, and 73 (16.8%) prefer not to. The details are shown in Table 2.

**Table 2:** Distribution of the study sample based on the COVID-19 infection status, beliefs, and education

		Education						
		Illiterate		Literate		Total		
		Count	Column N %	Count	Column N %	Count	Column N %	
Have you been infected with COVID-19?	No	200	82.3	148	77.5	348	80.2	P=0.2 (not significant)
	Yes	43	17.7	43	22.5	86	19.8	
Have any of your family members been infected with COVID-19?	No	138	56.8	97	50.8	235	54.1	P=0.2 (not significant)
	Yes	105	43.2	94	49.2	199	45.9	
Willing to take COVID-19 vaccines.	Strongly agree	7	2.9	26	13.6	33	7.6	P=0.001 (significant)
	Agree	40	16.5	26	13.6	66	15.2	
	Neutral	38	15.6	47	24.6	85	19.6	
	Disagree	61	25.1	55	28.8	116	26.7	
	Strongly disagree	97	39.9	37	19.4	134	30.9	
I recommend COVID-19 vaccines to my family members.	Strongly agree	40	16.5	42	22.0	82	18.9	-
	Agree	74	30.5	73	38.2	147	33.9	
	Neutral	106	43.6	69	36.1	175	40.3	
	Disagree	21	8.6	6	3.1	27	6.2	
	Strongly disagree	2	8.0	1	5.0	3	7.0	
I think I am not at risk to get a COVID-19 infection.	Strongly agree	15	6.2	13	6.8	28	6.5	P=0.58 (not significant)
	Agree	24	9.9	17	8.9	41	9.4	
	Neutral	137	56.4	95	49.7	232	53.5	
	Disagree	47	19.3	48	25.1	95	21.9	
	Strongly disagree	20	8.2	18	9.4	38	8.8	
Chances of contracting a COVID-19 infection.	Highly likely	25	10.3	21	11.0	46	10.6	P=0.01 (significant)
	Likely	53	21.8	69	36.1	122	28.1	
	Neutral	133	54.7	79	41.4	212	48.8	
	Unlikely	18	7.4	13	6.8	31	7.1	
	Highly unlikely	14	5.8	9	4.7	23	5.3	
If I caught a COVID-19 infection, I am sure it will be a subbing clinic.	Strongly agree	5	2.1	8	4.2	13	3.0	P=0.02 (significant)
	Agree	27	11.1	11	5.8	38	8.8	
	Neutral	140	57.6	96	50.3	236	54.4	
	Disagree	63	25.9	62	32.5	125	28.8	
	Strongly disagree	8	3.3	14	7.3	22	5.1	
I prefer to acquire immunity against COVID-19 naturally.	Strongly agree	30	12.3	25	13.1	55	12.7	P=0.6 (not significant)
	Agree	60	24.7	55	28.8	115	26.5	
	Neutral	115	47.3	76	39.8	191	44.0	
	Disagree	33	13.6	31	16.2	64	14.7	
	Strongly disagree	5	2.1	4	2.1	9	2.1	

Among the 434 unvaccinated elderly, most of them, 255 (58.8%), were unaware of COVID-19 vaccines, and the remaining 179 (41.2%) were aware of vaccines. However, only one-fifth, 92 (21.2%), believed that getting immunized with COVID-19 vaccines could protect against COVID-19 infection as well as prevent severe infection or death when infected. Nearly 304 (70%) of them were aware that COVID-19 vaccines would be administered through intramuscular injection. In terms of COVID-19 vaccines, one-third of the 129 participants (29.7%) said that vaccines could be given to children under the age of 12. Around 58% (253) expressed that they were not aware of whether to give to children under the age of 12 years. Nearly 22.6% (98) expressed that the body will produce immunity after taking COVID-19 vaccines. Higher-educated individuals had more knowledge, and the difference between educational categories was statistically significant. The details are shown in Table 3.

**Table 3:** Distribution of the study sample based on COVID-19 vaccine knowledge and education

COVID-19 vaccine knowledge		Education						
		Illiterate		Literate		Total		
		Count	Column N %	Count	Column N %	Count	Column N %	P-value
COVID-19 vaccine protects from COVID-19 infection.	Don’t know	179	73.7	76	39.8	255	58.8	p=0.001 (significant)
	No	25	10.3	62	32.5	87	20	
	Yes	39	16	53	27.7	92	21.2	
COVID-19 vaccine protects the vaccine receiver from getting a severe infection.	Don’t know	152	62.6	62	32.5	214	49.3	p=0.001 (significant)
	No	54	22.2	71	37.2	125	28.8	
	Yes	37	15.2	58	30.4	95	21.9	
									COVID-19 vaccination may protect other people who did not receive it.	Don’t know	203	83.5	104	54.5	307	70.7	0.001 (significant)
No	18	7.4	50	26.2	68	15.7											
Yes	22	9.1	37	19.4	59	13.6											
How many doses are needed to complete the vaccination in Saudi?	Don’t know	155	63.79	39	20.42	194	44.7	0.002 (significant)
	One dose	9	3.7	7	3.66	16	3.69	
	Two doses	30	12.35	125	65.45	155	35.71	
	Three doses	49	20.16	20	10.47	69	15.9	
COVID-19 vaccines are given by intramuscular injection.	Don’t know	156	64.2	38	12.6	194	44.7	p=0.04 (significant)
	No	15	6.17	11	1	26	5.99	
	Yes	72	29.63	142	74.35	214	49.31	
The COVID-19 vaccine does not have side effects.	Don’t know	93	38.3	28	14.7	121	27.9	p=0.001 (significant)
	No	113	46.5	140	73.3	253	58.3	
	Yes	37	15.2	23	12	60	13.8	
Children under 12 years of age can receive COVID-19 vaccines.	Don’t know	172	70.8	81	42.4	253	58.3	p=0.0001 (significant)
	No	24	9.9	28	14.7	52	12	
	YES	47	19.3	82	42.9	129	29.7	
COVID vaccine is highly recommended for above 12 years.	Don’t know	184	75.7	94	49.2	278	64.1	p=0.001 (significant)
	No	20	8.2	31	16.2	51	11.8	
	Yes	39	16	66	34.6	105	24.2	
COVID-19 vaccines stimulate our body to produce immunity.	Don’t know	208	85.6	113	59.2	321	74	p=0.001 (significant)
	No	7	2.9	8	4.2	15	3.5	
	Yes	28	11.5	70	36.6	98	22.6	

In terms of the perceptions of the COVID-19 vaccines, nearly 45.2% (192) perceived that COVID-19 vaccines are not fake, and 39 (09%) perceived that COVID-19 vaccines are of no use at all. Almost 168 (39%) of the participants expressed that vaccines were developed very quickly and approved on an emergency basis. Nearly one-third, 114 (26.1%), of the participants perceived that COVID-19 vaccines were promoted commercially by big companies. The details are shown in Table 4.

**Table 4:** The distribution of the study sample based on gender and perception toward COVID-19 vaccines

		Gender						
		Female		Male		Total		
		Count	Column N %	Count	Column N %	Count	Column N %	p-value
The COVID-19 vaccine is a fake vaccine and not a real vaccine.	Strongly agree	11	5.6	11	4.6	22	5.1	0.001 (significant)
	Agree	5	2.6	12	5.0	17	3.9	
	Neutral	114	58.2	89	37.4	203	46.8	
	Disagree	57	29.1	94	39.5	151	34.8	
	Strongly disagree	9	4.6	32	13.4	41	9.4	
	Total	196	100.0	238	100	434	100	
The COVID-19 vaccine was rapidly developed and approved.	Strongly agree	12	6.1	39	16.4	51	11.8	0.001 (significant)
	Agree	37	18.9	80	33.6	117	27.0	
	Neutral	134	68.4	101	42.4	235	54	
	Disagree	10	5.1	16	6.7	26	6.0	
	Strongly disagree	3	1.5	2	.8	5	1.2	
	Total	196	100	238	100	434	100	
The COVID-19 vaccine has a long-term harmful effect.	Strongly agree	41	20.9	44	18.5	85	19.6	0.10 (not significant)
	Agree	79	40.3	112	47.1	191	44.0	
	Neutral	67	34.2	64	26.9	131	30.2	
	Disagree	5	2.6	15	6.3	20	4.6	
	Strongly disagree	4	2.0	3	1.3	7	1.6	
	Total	196	100	238	100.0	434	100	
The COVID-19 vaccine is being promoted for commercial gains.	Strongly agree	11	5.6	33	13.9	44	10.1	0.001 (significant)
	Agree	20	10.2	50	21.0	70	16.1	
	Neutral	133	67.9	90	37.8	223	51.4	
	Disagree	24	12.2	53	22.3	77	17.7	
	Strongly disagree	8	4.1	12	5.0	20	4.6	
	Total	196	100	238	100	434	100	

One-quarter of the participants, 115 (26.5%), believed that COVID-19 vaccines protect others, and very few, 47 (10.8%), disagreed that they do not protect others. Most of the participants, 245 (56.4%), expressed that there is no conspiracy behind the COVID-19 vaccines. The education status agreed that there was no conspiracy behind the COVID-19 vaccines. The details are shown in Table 5.

**Table 5:** The distribution of the study sample based on education and beliefs toward COVID-19 vaccines

		Illiterate		Primary		Secondary		Bachelors		Total		p-value
		No	%	No	%	No	%	No	%	No	%	
It is important to get the COVID-19 vaccine to protect others.	Strongly agree	20	8	8	10	10	10	1	17	39	9	0.3 (not significant)
	Agree	39	16	14	17	22	22	1	17	76	18	
	Neutral	161	66	51	61	58	57	2	33	272	63	
	Disagree	20	8	10	12	9	9	1	17	40	9	
	Strongly disagree	3	1	1	1	2	2	1	17	7	2	
	Total	243	100	84	100	101	100	6	100	434	100	
I think there is a conspiracy behind the COVID-19 vaccine.	Strongly agree	7	3	1	1	0	0	0	0	8	2	0.3 (not significant)
	Agree	21	9	11	13	9	9	0	0	41	9	
	Neutral	89	37	20	24	28	28	3	50	140	32	
	Disagree	96	40	38	45	38	38	0	0	172	40	
	Strongly disagree	30	12	14	17	26	26	3	50	73	17	
	Total	243	100	84	100	101	100	6	100	434	100	
There is no harm in taking the COVID-19 vaccine.	Strongly agree	4	2	4	5	11	11	0	0	19	4	0.001 (significant)
													Agree	28	12	11	13	15	15	2	33	56	13
	Neutral	110	45	28	33	40	40	1	17	179	41	
	Disagree	63	26	35	42	29	29	2	33	129	30	
	Strongly disagree	38	16	6	7	6	6	1	17	51	12	
	Total	243	100	84	100	101	100	6	100	434	100	

Most of the participants, 346 (80%), revealed that vaccine centers were very accessible to them. In terms of vaccine availability and accessibility, 397 (91.5%) elderly mentioned that vaccines were available all the time. Most of the elderly, 408 (94%), opined that vaccine appointments were always available, and only six (1.4%) felt vaccine appointments were not available. The participants were mainly concerned with the safety of COVID-19 vaccines. Nearly 96 (22.1%) agreed that the benefits outweighed the side effects; 63 (14.5%) said the benefits outweighed the side effects; and the remaining 275 (63.4%) said they had no idea whether the benefits of taking the COVID-19 vaccine outweighed the side effects. After receiving COVID-19 vaccines, there is no need to follow the COVID-19 regulations; most of the participants, 317 (73%), disagreed and said that regardless of whether vaccines are taken or not, we must follow the COVID-19 regulations. For most of the elderly, the COVID-19 regulations are not relaxed even after they have received vaccines. They felt that vaccines were a waste of time, and there were side effects as well. The details are shown in Table 6.

**Table 6:** The distribution of the study participants based on the perception of the accessibility of COVID-19 vaccines

		Education					
		Illiterate		Literate		Total	
		Count	%	Count	%	Count	%
COVID-19 vaccine centers are accessible and near my home.	Strongly agree	125	51.4	131	68.6	256	59.0
	Agree	59	24.3	31	16.2	90	20.7
	Neutral	2	.8	3	1.6	5	1.2
	Disagree	43	17.7	19	9.9	62	14.3
	Strongly disagree	14	5.8	7	3.7	21	4.8
Is a COVID-19 vaccine appointment available to take the vaccine?	Strongly agree	190	78.2	157	82.2	347	80.0
	Agree	42	17.3	19	9.9	61	14.1
	Neutral	8	3.3	12	6.3	20	4.6
	Disagree	2	.8	2	1.0	4	9
	Strongly disagree	1	.4	1	.5	2	.5
The vaccine is not available and accessible.	Strongly agree	3	1.2	5	2.6	8	1.8
	Agree	11	4.5	2	1.0	13	3.0
	Neutral	8	3.3	8	4.2	16	3.7
	Strongly disagree	221	90.9	176	92.1	397	91.5
I feel the benefits of taking the COVID-19 vaccine outweigh the side effects.	Strongly agree	10	4.1	17	8.9	27	6.2
	Agree	35	14.4	34	17.8	69	15.9
	Neutral	162	66.7	113	59.2	275	63.4
	Disagree	27	11.1	19	9.9	46	10.6
	Strongly disagree	9	3.7	8	4.2	17	3.9
After getting the COVID-19 vaccine, I don’t need to follow the COVID rules.	Strongly agree	13	5.3	11	5.8	24	5.5
	Agree	27	11.1	15	7.9	42	9.7
	Neutral	34	14.0	17	8.9	51	11.8
	Disagree	79	32.5	46	24.1	125	28.8
	Strongly disagree	90	37.0	102	53.4	192	44.2

Out of 434 participants, 308 (70.9%) were afraid of the harmful side effects of vaccines. Fear of side effects was nearly equal in both males and females, 164 (68.9%) and 144 (73.5%), respectively, but the difference was not statistically significant. Nearly 200 (46%) expressed that COVID-19 vaccines are not safe, with 104 (53%) being female and 96 (40%) being male. The gender difference in vaccine safety was statistically significant (p=0.04). Only 85 (19.6%) felt that COVID-19 vaccines are effective, 88 (20.3%) felt that they are not effective, and most of the participants, 261 (60.1%), were undecided about whether vaccines are effective or not. The difference between vaccine effectiveness knowledge and gender was statistically significant (p=0.001). Most of the elderly females, 122 (62%), were afraid of injections, whereas only 70 males (29.4%) were afraid of injections. The difference between gender and fear of injection was statistically significant (p=0.001). The details are shown in Table 7.

**Table 7:** The participants' responses regarding their knowledge about COVID-19 vaccination (n = 434 participants) ICU: intensive care unit

Knowledge items related to vaccination	Correct		Incorrect		Do not know	
Against COVID-19.	No.	%	No.	%	No.	%
Routes of vaccine administration.	304	70.0	8	1.8	122	28.1
It is recommended to vaccinate those aged >12 years.	105	24.2	51	11.8	278	64.1
Vaccination stimulates antibodies against COVID-19.	98	22.6	15	3.5	321	74.0
Vaccination protects against admission to ICU.	95	21.9	125	28.8	214	49.3
Vaccination protects well against COVID-19.	92	21.2	87	20.0	255	58.8
Vaccines have side effects.	60	13.8	253	58.3	121	27.9
Vaccination can protect unvaccinated contacts.	59	13.6	68	15.7	307	70.7
No. of scheduled doses of the vaccines.	55	12.7	185	42.6	194	44.7
Children under 12 years should be vaccinated.	52	12.0	129	29.7	253	58.3

Tables 8 and 9 show that the most negative attitudes toward COVID-19 infection and vaccination were associated with participants preferring to be infected rather than vaccinated (12.7% strongly agreed and 26.5% agreed) and having no time to get vaccinated (7.4% strongly agreed and 20.5% agreed). On the other hand, The participants' lowest positive attitudes were related to vaccine safety, i.e., not being harmful, and effectiveness in COVID-19 prevention (5.3% strongly agreed and 15.9% agreed); the benefits of taking vaccines outweigh its risks (6.2% strongly agreed and 15.9% agreed) and taking vaccine if available (7.6% strongly agreed and 15.2% agreed).

**Table 8:** The participants' attitudes toward COVID-19 infection and vaccination

Attitude items	Strongly agree		Agree		Neutral		Disagree		Strongly disagree	
	No.	%	No.	%	No.	%	No.	%	No.	%
Negative attitude										
I am not at any risk to get a severe infection	28	6.5	41	9.4	232	53.5	95	21.9	38	8.8
If I get infected, it will be mild.	13	3	38	8.8	236	54.4	125	28.8	22	5
I prefer to be infected than to be vaccinated.	55	12.7	115	26.5	191	44	64	14.7	9	2.1
It is important to get vaccinated to protect others.	7	1.6	76	17.5	272	62.7	40	9.2	39	9
I think there is a harmful conspiracy behind vaccination.	8	1.8	41	9.4	140	32.3	172	39.6	73	16.8
No need to follow the preventive measures after being vaccinated.	24	5.5	42	9.7	51	11.8	125	28.8	192	44.2
Vaccination is neither available nor accessible.	8	1.8	13	3	16	3.7	0	0	397	91.5
I have no time to get vaccinated.	32	7.4	89	20.5	30	6.9	166	38.2	117	27
Positive attitude										
I will take the vaccine if available.	33	7.6	66	15.2	85	19.6	116	26.7	134	30.9
I will advise others to be vaccinated.	82	18.9	147	33.9	175	40.3	27	6.2	3	0.7
There is no harm in being vaccinated	19	4.4	56	12.9	179	41.2	129	29.7	51	11.8
I think the vaccines are available free of charge.	359	82.7	57	13.1	6	1.4	12	2.8	0	0
An appointment to take the vaccine is available.	347	80	61	14.1	20	4.6	4	0.9	2	0.5
Vaccine centers are available.	256	59	90	20.7	5	1.2	62	14.3	21	4.8
The benefits of taking the vaccine outweigh the risks.	27	6.2	69	15.9	275	63.4	46	10.6	17	3.9
The vaccine is safe and effective.	23	5.3	69	15.9	274	63.1	56	12.9	12	2.8

**Table 9:** Concerns associated with participants’ refrain from receiving vaccination against COVID-19

Concerns	No.	%
Religious restrictions	10	2.3
Cultural restrictions	14	3.2
Unavailability of the vaccines	21	4.8
COVID-19 infection is not dangerous	33	7.6
The vaccine is fake	39	9.0
Concerns against vaccination	61	14.1
Not effective	88	20.3
The vaccines were developed for commercial reasons	114	26.3
No time to get the vaccine	121	27.9
No sufficient studies about the vaccines	125	28.8
Having no information about the vaccines	132	30.4
The vaccines were hastily developed	168	38.7
General fear of injection	192	44.2
The vaccines are not safe	200	46.1
Alarming information about the vaccines	247	56.9
The vaccines cause prolonged harms	276	63.6
The vaccines have serious side effects	309	71.2
Strong belief in traditional remedies	343	79.0

## Discussion

About 26.1% of the participants perceived that COVID-19 vaccines were promoted commercially by big companies. Although awareness regarding programs for vaccination against COVID-19 has been globally created, vaccination hesitancy, in the form of a delay in acceptance or even refusal of vaccination despite the availability of vaccination services, has impeded its implementation worldwide. Therefore, vaccine hesitancy has been identified, even before the COVID-19 pandemic, as one of the top 10 threats to global health [15]. Despite the fact that several studies on knowledge about COVID-19 vaccination have been conducted in other countries [16,17], no study has been conducted in Saudi Arabia. To the best of our knowledge, this is the first study to examine the level of COVID-19 vaccine knowledge among unvaccinated elderly people in Saudi Arabia. The present study aimed to find out the knowledge, attitudes, and concerns among the unvaccinated elderly over the age of 60 years about COVID-19 in the Aseer Region, Saudi Arabia. Our study revealed that the knowledge grade of most participants about COVID-19 vaccination was poor. Apart from their knowledge of the routes of COVID-19 vaccine administration, which 70% of respondents correctly answered, they responded to all other addressed knowledge statements with "I do not know" or incorrect answers. Participants' least correct answers concerned vaccine side effects, unvaccinated contact protection, the number of scheduled vaccine doses, and whether children under the age of 12 should be vaccinated.

Our study showed that only about one-third of the participants considered the chances of getting infected with COVID-19 to be likely, while almost half of them were uncertain regarding their chance of being infected. Some participants' most negative attitudes toward COVID-19 infection and vaccination were associated with a preference to be infected rather than vaccinated and a lack of time to get vaccinated. On the other hand, participants' lowest positive attitudes were related to there being no harm in being vaccinated; vaccine safety in terms of not being harmful; effectiveness in preventing COVID-19 infection; benefits outweighing risks; and taking vaccines if available.

Moreover, our study revealed that, although almost half of the elderly participants had a positive family history of COVID-19 infection and 19.8% of them had been previously infected with COVID-19, all of them remained unvaccinated against COVID-19. Their highest concerns that are associated with their vaccine hesitancy are the alarming information they receive about vaccines, their misbelief that vaccines cause prolonged harm or serious side effects, and their current strong belief in traditional remedies. Other associated concerns were related to vaccine safety, their general fears toward injections, and the fact that the COVID-19 vaccines were hastily developed. The highest number of participants' concerns was observed among participants relying on social media as their main source of knowledge regarding COVID-19 vaccines.

Alam et al. [18] stated that the public’s attitudes toward vaccination may be an expression of support or hesitancy. Examples of negative attitudes toward vaccines include the unwillingness or deferral to receive vaccinations, which are major barriers to controlling the COVID-19 pandemic. Any concerns, misinformation, or rumors can easily propagate through social media and can be instrumental in developing a negative attitude among the target population. This can lead to vaccine hesitancy or denial of receiving vaccines despite their availability. Therefore, it is important to study the factors that influence people’s attitudes toward COVID-19 vaccination or those associated with hesitancy. A proper understanding of people’s attitudes can be useful in overcoming vaccine hesitancy, which can aid in the design of health education and social marketing efforts to remove the gaps in knowledge and overcome their concerns and misconceptions. Pertwee et al. noted that, at the individual level, the factors influencing vaccination include past negative experiences related to vaccination, general misbeliefs and attitudes about health and prevention, perceived risks and benefits, and social norms concerning vaccines [19]. Lazarus et al. emphasized that concerns about the side effects constitute an important barrier to vaccine acceptance among the Saudi population [20]. In Saudi Arabia, Marzo et al. reported that participants showed a positive and optimistic attitude toward COVID-19 [21]. Most participants reported that the virus can be successfully controlled, while 97% were convinced that the Saudi government will easily control the pandemic. The public's positive attitudes and high confidence in the control of COVID-19 can be explained by the government’s unprecedented actions and prompt response in taking rigorous control and precautionary measures against COVID-19 to protect citizens and ensure their well-being. These measures included the lockdown, the suspension of all domestic and international flights, prayer at mosques, schools, and universities, and the national curfew imposed on citizens [21].

Our study showed that participants’ knowledge grades regarding COVID-19 vaccination were poorest among those aged above 80 years, among the illiterate, the unemployed, and current smokers, in addition to those whose main source of knowledge was the mass media. Moreover, the number of concerns among the elderly who were unvaccinated was significantly higher among those aged less than 70 years, highly educated, employed, and current smokers. Poor knowledge among our participants may be attributed to the finding that participants' sources of information regarding COVID-19 vaccination were mostly unreliable, even among those highly educated. Their main sources of information were the mass media, followed by their friends, families, or neighbors, and social media, but they did not include healthcare providers.

Limitations

These limitations include potential reliance on self-reported data, the lack of a detailed description of the knowledge assessment tool, the absence of longitudinal data, failure to account for external factors, limited scope, and generalizability concerns. Addressing these limitations would enhance the study's validity and provide a more comprehensive understanding of vaccine acceptance among unvaccinated older adults in the region.

## Conclusions

Most unvaccinated elderly people in the Aseer Region, Saudi Arabia, know little about COVID-19 vaccination, especially those over 80, illiterate, unemployed, and smokers. Mass media is their main source of information about COVID-19 vaccination, followed by their friends, families, neighbors, and social media. Participants' biggest concerns are alarming vaccine information, vaccines' long-term side effects, and their strong belief in traditional remedies. Vaccines are unsafe and develop quickly, and people fear injections. The study recommends intensifying educational activities through mass media, addressing misinformation on social media, and implementing tailored interventions to improve knowledge and address concerns among unvaccinated older adults.
